# Accuracy and Completeness of ChatGPT-Generated Information on Interceptive Orthodontics: A Multicenter Collaborative Study

**DOI:** 10.3390/jcm13030735

**Published:** 2024-01-27

**Authors:** Arjeta Hatia, Tiziana Doldo, Stefano Parrini, Elettra Chisci, Linda Cipriani, Livia Montagna, Giuseppina Lagana, Guia Guenza, Edoardo Agosta, Franceska Vinjolli, Meladiona Hoxha, Claudio D’Amelio, Nicolò Favaretto, Glauco Chisci

**Affiliations:** 1 Orthodontics Postgraduate School, Department of Medical Biotechnologies, University of Siena, 53100 Siena, Italy; tiziana.doldo@unisi.it (T.D.); linda.cipriani1@gmail.com (L.C.); 2 Oral Surgery Postgraduate School, Department of Medical Biotechnologies, University of Siena, 53100 Siena, Italy; stefano.parrini@unisi.it; 3 Orthodontics Postgraduate School, University of Ferrara, 44121 Ferrara, Italy; 4 Orthodontics Postgraduate School, University of Cagliari, 09121 Cagliari, Italy; montagnadssalivia@gmail.com; 5 Orthodontics Postgraduate School, “Sapienza” University of Rome, 00185 Rome, Italy; giuseppina.lagana@unicamillus.org; 6 Orthodontics Postgraduate School, University of Milano, 20019 Milan, Italy; 7 Orthodontics Postgraduate School, University of Torino, 10024 Turin, Italy; 8 Orthodontics Postgraduate School, University of Roma Tor Vergata, 00133 Rome, Italy; f.vinjolli@unizkm.al; 9 Orthodontics Postgraduate School, “Cattolica” University of Rome, 00168 Rome, Italy; meladionahoxha@gmail.com; 10 Orthodontics Postgraduate School, University of Chieti, 66100 Chieti, Italy; thecrodin@gmail.com; 11 Orthodontics Postgraduate School, University of Trieste, 34100 Trieste, Italy

**Keywords:** artificial intelligence, artificial bot, ChatGPT, methodology, orthodontics, interceptive orthodontics

## Abstract

**Background**: this study aims to investigate the accuracy and completeness of ChatGPT in answering questions and solving clinical scenarios of interceptive orthodontics. **Materials and Methods**: ten specialized orthodontists from ten Italian postgraduate orthodontics schools developed 21 clinical open-ended questions encompassing all of the subspecialities of interceptive orthodontics and 7 comprehensive clinical cases. Questions and scenarios were inputted into ChatGPT4, and the resulting answers were evaluated by the researchers using predefined accuracy (range 1–6) and completeness (range 1–3) Likert scales. **Results**: For the open-ended questions, the overall median score was 4.9/6 for the accuracy and 2.4/3 for completeness. In addition, the reviewers rated the accuracy of open-ended answers as entirely correct (score 6 on Likert scale) in 40.5% of cases and completeness as entirely correct (score 3 n Likert scale) in 50.5% of cases. As for the clinical cases, the overall median score was 4.9/6 for accuracy and 2.5/3 for completeness. Overall, the reviewers rated the accuracy of clinical case answers as entirely correct in 46% of cases and the completeness of clinical case answers as entirely correct in 54.3% of cases. **Conclusions**: The results showed a high level of accuracy and completeness in AI responses and a great ability to solve difficult clinical cases, but the answers were not 100% accurate and complete. ChatGPT is not yet sophisticated enough to replace the intellectual work of human beings.

## 1. Introduction

The recent mass diffusion of the use of artificial intelligence in all sectors has increased and the software development ChatGPT has been implemented. Chat-based generative pre-trained transformer (ChatGPT) [1] is an advanced artificial intelligence (AI) language model developed by Open Artificial Intelligence (San Francisco, CA, USA). The first version was released in November 2022 and quickly became the fastest-growing application in history, counting 100 million active users as of February 2023 [2]. It is based on the GPT-4 architecture and is designed to understand and generate human-like text responses in a conversation. ChatGPT has been trained on a wide range of data sources, making it capable of providing information, answering questions, and engaging in conversation across various topics.

Furthermore, new lines of development have recently appeared in artificial intelligence alternatives to ChatGPT. Also, AI has spread commonly as a research tool for gaining knowledge, including on healthcare questions and answers. The use of ChatGPT in the healthcare industry has substantial potential. Initially, it can assist healthcare practitioners in diagnosing medical conditions by examining patient symptoms, medical history, and other relevant data, leading to faster and more accurate diagnoses. Additionally, ChatGPT can contribute to the synthesis and analysis of extensive medical literature, ultimately possibly resulting in the identification of new treatments, medications, or a deeper comprehension of diseases [3,4,5,6,7,8]. Further, ChatGPT has the potential to serve as an additional educational resource for medical students and professionals by offering explanations, responding to queries, and assisting in the revision of medical concepts [9,10]. Furthermore, it could be utilized to empower virtual assistants that aid patients in managing their health, providing details about medications and treatments, and addressing typical health-related inquiries. These potential uses showcase the positive influence that ChatGPT could have on healthcare [11,12,13,14,15].

These applications come with several limitations and ethical considerations, like credibility [16] and plagiarism [17,18]. ChatGPT adheres to the EU’s AI ethical guidelines, which emphasize the crucial role of human oversight, technical robustness and safety, privacy, and data governance [19]. Before implementing ChatGPT, the potential limitations and ethical considerations need to be thoroughly assessed and addressed [20]. 

The authors conducted research to find any articles on ChatGPT and interceptive orthodontics, but to date, there are no articles that analyze the use of ChatGPT in interceptive orthodontics. The authors chose interceptive orthodontics as it represents a great matter of interest for orthodontics residents and dental students and represents the majority of the treatments in Italian national health services. For this reason, this research was born to judge the accuracy and completeness of answers to open questions and clinical cases in the field of interceptive orthodontics.

## 2. Materials and Methods

In March 2023, a research group was established to investigate the potential applications of AI platforms in interceptive orthodontics. This collaborative group recruited ten specialized orthodontists with a postgraduate degree from ten different Italian orthodontics postgraduate schools, encompassing a wide range of expertise and experiences. The postgraduate orthodontics schools that participated to this study are the University of Siena, the University of Ferrara, the University of Cagliari, “Sapienza” University of Rome, the University of Milano, the University of Torino, the University of Roma Tor Vergata, the University “Cattolica” University of Rome, the University of Chieti, and the University of Trieste. The research purpose was to explore the usefulness and reliability of AI platforms in various interceptive orthodontics-related tasks and decision-making processes. This study design did not require the approval of an ethics committee, as per Italian legislation on clinical investigations at the time of the study, as it did not involve patients.

For this study, the participating researchers were strategically divided into seven working groups based on their respective areas of expertise. This division was designed to ensure comprehensive coverage of the diverse areas of interceptive orthodontics, such as II class malocclusion; III class malocclusion; cephalometric analysis; dental inclusion; open bite; atypical swallowing; deep bite. Each group was tasked with leveraging their specific expertise to develop a set of three open-ended clinical questions that would challenge the AI platform. The difficulty level of each question was subjectively assessed by the researchers, based on their expertise and understanding of the subject matter.

Furthermore, the researchers prepared a series of 7 comprehensive clinical scenarios, drawing inspiration from real cases that were assessed at their respective affiliated orthodontics departments. Each scenario included detailed patient histories, patient age, and signs and symptoms exhibited by the patients, with the primary objective of evaluating the AI platform’s accuracy and completeness in evaluating the patient clinical context and subsequently proposing a suitable diagnostic pathway.

Upon completion, all questions and clinical cases were evaluated by the entire research group that revised and approved the text based on the quality and completeness of the questions to submit appropriate and correct challenges to the AI platform. The complete set of clinical questions and scenarios is presented in Table 1 and Table 2.

To ensure consistency in the study, a single researcher inserted all of the questions and clinical scenarios into the ChatGPT version 4 on 21 April 2023. For each question, the researcher instructed the AI to provide specific answers, considering available guidelines before submitting the question. Further, for the clinical scenarios, the AI was requested to identify the most probable diagnosis.

All responses obtained through this process were recorded and provided to the researchers for evaluation. In this way, each answer was subsequently evaluated by ten different researchers. As described by Johnson et al. [21], the researchers assessed the accuracy and completeness of the open-ended answers and the clinical scenarios using two predefined scales. For accuracy, a six-point Likert [22] scale was employed, with 1 representing a completely incorrect response, 2 denoting more incorrect than correct elements, 3 indicating an equal balance of correct and incorrect elements, 4 signifying more correct than incorrect elements, 5 representing nearly all correct elements, and 6 being entirely correct. As for completeness, a three-point Likert scale was used: 1 stood for an incomplete answer that only addressed some aspects of the question with significant parts missing or incomplete, 2 represented an adequate answer that addressed all aspects of the question and provided the minimum information required for completeness, and 3 denoted a comprehensive response that covered all aspects of the question and offered additional information or context beyond expectations. 

### Statistical Analyses

Statistical analyses were performed using Jamovi version 2.3.18.0, a freeware and open statistical software available online at www.jamovi.org accessed on 1 August 2023 [23]. Categorical variables are reported in numerals and percentages of the total. The Shapiro–Wilk test was used to verify the normality of the distribution. The level of statistical significance was set at *p* < 0.05 with a 95% confidence interval. Interrater reliability was calculated with the α *Cronbach* using jamovi.org.

## 3. Results

**Figure 1:**
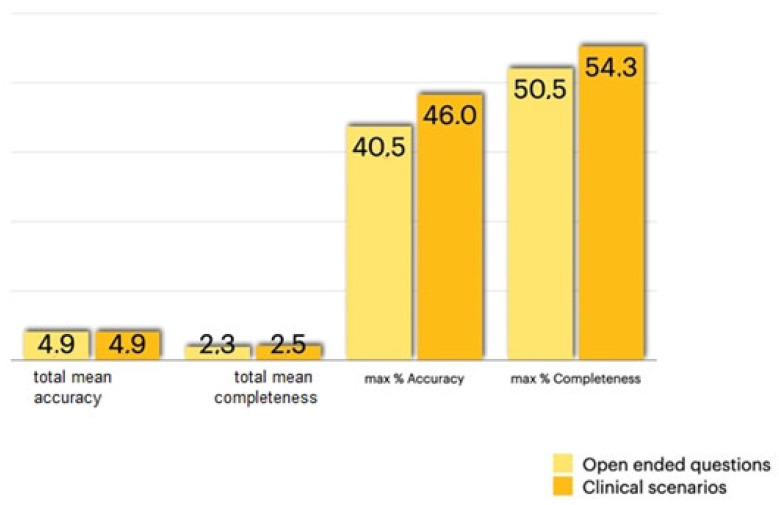
Total mean accuracy, total mean completeness, maximum accuracy, and maximum completeness of the open-ended questions and clinical scenarios.

For the open-ended questions, the overall median score was 4.9/6 for accuracy and 2.4/3 for completeness. Overall, the reviewers rated the open-ended answers as entirely correct in 40.5% of cases and 50.5% of cases as comprehensive and covering all aspects of the question (Figure 1).

As for the clinical cases, the overall median score was 4.9/6 for accuracy and 2.5/3 for completeness. Overall, the reviewers rated the clinical case answers as entirely correct in 46% of cases and 54.3% of cases as comprehensive and covering all aspects of the question (Figure 1). 

For the open-ended questions, α Cronbach was 0.640 for accuracy and 0.524 for completeness; for the clinical scenarios, it was 0.487 for accuracy and 0.636 for completeness.

## 4. Discussion

The extensive use of ChatGPT in healthcare may lead to many strength and weakness points [24]. One of the main strengths of ChatGPT is its ability to provide personalized health information and support to individuals. One of the main weaknesses is the potential for misinterpretation or miscommunication, as language models may not always accurately understand the nuances of human language and context [25,26]. One of the most important aspects to assess when dealing with AI is related to ethics. ChatGPT processes and preserves confidential health data, including personal information and medical records entered by patients in the chat box [27]. Guaranteeing the privacy and safety of this information is crucial to avoid non-authorized access, data violations, or identity fraud [28].

Regarding AI and ethics, the European Union in 2019 developed its ethical code [19] that contains guidelines on the use and development of artificial intelligence systems. In 2022, the University of Siena was the first Italian university to define its own guidelines on the use of ChatGPT by students and lecturers [29]. Finally, in October 2023, also the World Health Organization has expressed its views on the ethics of AI [30].

It is very interesting to point out that prior research has investigated the accuracy of information generated by ChatGPT in the field of head and neck and oromaxillofacial surgery. Mago et al. [31], in July 2023, reported that GPT-3 gave 100% accuracy in describing radiographic landmarks in oral and maxillofacial radiology, but regardless, GPT-3 cannot be considered a pillar for reference because it is less attentive to details and could give inaccurate information. In August 2023, Vaira et al. [32] conducted a multicenter collaborative study on the accuracy of ChatGPT-generated information on head and neck oromaxillofacial surgery. In their study, the authors reported that the reviewers rated the answers as entirely or nearly entirely correct in 87.2% of cases and as comprehensive and covering all aspects of the question in 73% of cases. But in any case, they concluded that AI is not a reliable support for making decisions regarding head–neck surgery. The values obtained by Vaira et al. are higher than in our research, but it must be considered that in our study, we considered only the answers entirely correct for the calculation of the percentage of accuracy and completeness.

To date, there are no scientific articles in the literature highlighting the use of ChatGPT for interceptive orthodontics. As far as orthodontics is concerned, Subramanian et al. [33], in their narrative review, affirm that AI is a promising tool for facilitating cephalometric tracing in routine clinical practice and analyzing large databases for research purposes. In the present study, we asked the artificial intelligence three questions about cephalometry: question number 7: “What is the cephalometric divergence value in skeletal Class II?”; question number 8: “What is cephalometric tracing in orthodontics?”; question number 9: “What is the average value of the SNA angle in orthodontics?”. The median for answer number 7 was 4/6 points for accuracy and 2/3 for completeness The median for answer number 8 was 5.4/6 points for accuracy and 2.6/3 points for completeness. Lastly, the median for answer number 9 was 5.3/6 points for accuracy and 2.4/3 points for completeness.

Tanaka et al., have reported that ChatGPT has proven effective in providing quality answers related to clear aligners, temporary anchorage devices, and digital imaging within the context of interest of orthodontics [34]. Tanaka et al.’s study and the present study are very similar, but in the present study, a group of ten orthodontists with a postgraduate degree from ten different Italian orthodontics postgraduate schools gave their evaluation, while Tanaka et al.’s research group was formed of five general orthodontists. 

Duran et al., in their paper, report that ChatGPT provides highly reliable, high-quality, but challenging-to-read information related to cleft lip and palate (CLP) [35] and that the information obtained must be verified by a qualified medical expert. We agree with the last statement, as in the present study, ChatGPT affirms in many answers that it is not an orthodontist, and it recommends consulting with an expert orthodontist to solve the problem or decide on the best type of appliance needed for that specific case. So, ChatGPT, in many instances, gives answers but never recommends following only its instructions. 

Gonzales et al. [36], in their review, affirm that AI technology may determine the cervical vertebral maturation stage and obtain the same results as expert human observers. Moreover, AI technology may improve the diagnostic accuracy for orthodontic treatments, helping the orthodontist work more accurately and efficiently. The authors of the present study cannot agree with them, as in the present research results, the ten specialized orthodontists never rated the answers as totally accurate and complete, even if the total median of accuracy was 4.9/6 points for both open-ended questions and the clinical cases, the maximum percentage of accuracy was 46%, and the maximum percentage of completeness was 54.3%. 

Ahmed et al. affirm that their study validated the high potential for developing an accurate caries detection model that will expedite caries identification, assess clinician decision making, and improve the quality of patient care [37]. According to the authors of this study, the evaluation of X-rays is a static matter, whereas a comprehensive answer to an open question or a diagnosis and orthodontic treatment setting of a clinical case is an aspect for which AI is not yet ready. For this reason, even if Gonzales and Ahmed affirm that AI may help the dentist or the orthodontist, in our opinion, AI is not able to help the orthodontist concerning the questions it was asked about interceptive orthodontics.

Strunga et al. [38], in their scoping review, affirm that AI is an efficient instrument to manage orthodontic treatment from diagnosis to retention and patients find the software easy to use while clinicians can diagnose more easily. In our opinion, ChatGPT should not be a tool for patients because the answers it gave were not 100% accurate and complete. Moreover, ChatGPT has no legal responsibilities. So, ChatGPT should not be considered an instrument to help the orthodontist to make an orthodontic diagnosis or to choose the better treatment. The software failed to show flair and originality and emphasized in several answers that the diagnosis should be entrusted to an orthodontic specialist. Vishwanathaiah et al. [39], in their review, report that AI cannot be a replacement for dental clinicians/pediatric dentists. On the basis of the present research, AI did not report bibliographic references to support the answers. Some of the open-ended answers given by ChatGPT may appear incomplete but it could be due to the fact that the chatbot did not have the context for those questions. It should be interesting in future research to give more context and ask for a second answer.

The authors of this multicenter collaborative study are aware that the present study has some limitations due to the fact that ten specialized orthodontists is a preliminary number, but the value is that these ten specialists belong to ten different Italian universities. Further, ChatGPT is not a professor or an expert that independently understands the nuances of orthodontics; it is a tool that adapts its responses based on the information and context provided by the user. The questions should be written in a detailed, clear, and accurate way. The chat interface offers the user the opportunity to refine and direct the conversation for more precise and structured responses. In our study, only the first response given by ChatGPT was considered good.

So, for future perspectives, we support the need for a larger multicenter collaborative study formed by more than ten specialized orthodontists belonging to different universities and another study formed by both general orthodontists and specialized orthodontists in order to evaluate with a more comprehensive vision the output of information produced by artificial intelligence. Further, future research should compare the answers from ChatGPT and human students and postgraduates in order to evaluate and compare the differences.

Nonetheless, with the proper precautions in place, ChatGPT can be considered an interesting and very promising technology. The results of this study have clinical relevance as a report on new information regarding ChatGPT and orthodontics and could impact patient and parent education about interceptive orthodontics. It is a digital tool that can be useful at work as a starting point for further research, but it is not yet sophisticated enough to replace the intellectual work of human beings. There is a need to implement AI to achieve the absolute accuracy and completeness of its answers.

## 5. Conclusions

Ten specialized orthodontists from ten Italian postgraduate orthodontics schools (Siena, Ferrara, Sacro Cuore, Milan, Trieste, Tor Vergata, Turin, Cagliari, Chieti, and Cattolica) developed 21 clinical open-ended questions and 7 clinical cases about interceptive orthodontics. The results demonstrated a high level of accuracy and completeness in the AI’s answers and a strong ability to resolve complex clinical scenarios, but at the date of the present study, ChatGPT cannot be considered as a substitute for a doctor and should not be a tool for patients because the answers it has given have not been 100% accurate and complete. Numerous ethical concerns will need to be addressed and resolved in the future, especially due to the fact that ChatGPT has no legal responsibility. ChatGPT is not merely a question-and-answer tool like a Google search bar, but a sophisticated AI that requires careful and informed interaction to yield the best results. So, it should be an aspect to be considered for further research in this area.

### Disclaimer

In the guidelines on the use of ChatGPT laid down by the University of Siena [29], article 7 requires that “the authors of publications, degree and doctoral theses, dissertations or other writings in which the contribution of each author is determined, must clearly and specifically indicate whether and to what extent they have used artificial intelligence technologies such as ChatGPT (or other LLM) in the preparation of their manuscripts and analyses”. 

So, we can declare that artificial intelligence, namely, ChatGPT, was used only for the answers given to the 21 open-ended questions and the 7 clinical cases. Thus, the questions were formulated by the orthodontic specialists, and only the answers were given by ChatGPT; statistical analysis of the data was entirely performed directly by us through the site Jamovi.org.

## Figures and Tables

**Table 1:** The 21 open-ended questions submitted to ChatGPT and the answers obtained. Then, the accuracy and completeness mean score and Shapiro–Wilk p score were calculated for each answer obtained. Open-ended questions and answers given by ChatGPT-4.

Category	Question	ChatGPT-4 Answer	Accuracy Mean Score (Range 1–6)	Completeness Mean Score (Range 1–3)	Accuracy Shapiro–Wilk p Score	Completeness Shapiro–Wilk p Score
	1. What is the etiology of Class II malocclusion in orthodontics?	The exact etiology of Class II malocclusion is not fully understood, but there are several contributing factors that have been identified. These include genetics, developmental issues, environmental factors, and habits such as thumb-sucking. In many cases, Class II malocclusion is caused by a discrepancy between the size of the upper and lower jaws, resulting in the upper jaw being too far forward relative to the lower jaw. Other contributing factors may include missing or impacted teeth, premature loss of primary teeth, and injuries or trauma to the jaw or teeth. Treatment for Class II malocclusion may include orthodontic appliances, such as braces or aligners, or in some cases, surgery may be required to correct the underlying jaw discrepancy.	4.70	2.50	0.006	<0.001
II class malocclusion	2. What is the timing of Class II malocclusion treatment in orthodontics?	The timing of Cl II malocclusion treatment in orthodontics depends on the severity of the malocclusion and the age of the patient. Generally, treatment can begin around the age of seven or eight, when the first permanent molars have erupted and the orthodontist can evaluate the bite and the growth pattern of the jaw. However, in some cases, treatment may be delayed until adolescence, when all permanent teeth have erupted and growth is complete. The orthodontist will assess each patient individually and determine the best timing for treatment based on their specific needs.	4.40	2.20	0.445	0.012
II class malocclusion	3. What is the twin block in orthodontics?	The Twin Block is a type of functional appliance used in orthodontics to correct the bite of an individual in the growing stage, typically during pre-adolescence. The twin block appliance consists of two removable parts or blocks (one for the upper jaw and one for the lower jaw) that work together to realign the teeth and jaw. The appliance is designed to fit securely over the teeth and is worn for a specific period of time as recommended by the orthodontist. The Twin Block is often used to correct an overbite or underbite and is usually worn for several months to a year, depending on the individual case.	4.30	2.30	0.012	0.015
III class malocclusion	4. What is the etiology of Class III malocclusion in orthodontics?	The etiology of Class III malocclusion in orthodontics can be multifactorial and may involve both genetic and environmental factors. Some common causes include: Genetic factors: Class III malocclusion has been found to have a genetic component, which means that it can be inherited from parents. Abnormal growth of the jaws: An abnormal growth of the lower jaw or a deficient growth of the upper jaw can lead to a Class III malocclusion. Premature loss of teeth: When a patient loses primary or permanent teeth prematurely, it can affect the alignment of the remaining teeth, leading to malocclusion. Habits: Habits such as thumb sucking, tongue thrusting, and mouth breathing can contribute to the development of a Class III malocclusion. Skeletal trauma: Trauma to the jaw or face can cause a Class III malocclusion. Cleft lip and palate: Patients with cleft lip and palate are more likely to have a Class III malocclusion. Other medical conditions: Some medical conditions such as sleep apnea, hormonal imbalances, and craniofacial syndromes may contribute to the development of a Class III malocclusion.	5.10	2.70	0.019	<0.01
III class malocclusion	5. What is the timing of Class III malocclusion treatment in orthodontics?	The timing of Class III malocclusion treatment in orthodontics depends on several factors, including the severity of the malocclusion, the age of the patient, and the preference of the orthodontist. For mild cases, treatment can begin as early as age 7 or 8, while for more severe cases, treatment may need to be postponed until facial growth has slowed down, typically between the ages of 12 and 14. Early treatment can include the use of functional appliances or partial braces, while comprehensive orthodontic treatment with full braces or clear aligners may be needed in more severe cases. Ultimately, the best timing for Cl III malocclusion treatment will be determined by an orthodontist who evaluates the patient’s specific needs and recommends the appropriate course of treatment.	3.70	2.10	0.067	0.004
III class malocclusion	6. How is the Delaire mask made in orthodontics?	The Delaire mask, also known as the facemask or reverse-pull headgear, is typically made up of several components: Metal framework: A custom-made metal frame that fits over the patient’s head and attaches to their teeth or braces. Elastic bands: Elastic bands are attached to the facial part of the apparatus and are used to pull the upper jaw forward. Chin cup: A chin cup is often used as a way to anchor the elastic bands to the lower jaw. The process of making a Delaire mask usually involves creating a custom metal frame using a mold of the patient’s head and measuring the distance between the attachment points on the teeth. The elastic bands and chin cup are then attached to the frame and adjusted to apply the appropriate amount of force to promote the desired movement of the upper jaw. It’s essential to consult a trained orthodontist or dentist to ensure the proper fitting and adjustment of the Delaire mask, as improper use can lead to discomfort or pain for the patient.	5.30	2.50	<0.001	<0.001
Cephalometrics	7. What is the cephalometric divergence value in skeletal Class II?	The cephalometric divergence value in skeletal Class II can vary depending on the specific measurements being used and the severity of the skeletal discrepancy. However, in general, a higher cephalometric divergence value (indicating increased vertical skeletal growth) is often associated with Class II skeletal patterns, particularly in cases with an exaggerated overbite or excessive vertical facial height.	4.00	2.00	0.157	0.022
Cephalometrics	8. What is cephalometric tracing in orthodontics?	Cephalometric tracing in orthodontics is a diagnostic tool used to evaluate the position of the jaws and teeth, and to help plan orthodontic treatment. It involves creating line drawings, or tracings, of X-ray images of a patient’s head and neck in order to analyze the relationship of the bones and teeth. The tracing can show measurements of the teeth, the angle of the jaw, and other aspects of facial structure that can help guide orthodontic treatment. This technique is often used in conjunction with other diagnostic tools, like dental casts and photographs, in order to fully evaluate the needs of a patient and create an effective treatment plan.	5.40	2.60	<0.001	<0.011
Cephalometrics	9. What is the average value of the SNA angle in orthodontics?	The average value of the SNA angle in orthodontics is approximately 82 degrees.	5.30	2.40	0.015	0.008
Dental Inclusion	10. What is the prevalence of dental inclusion in mixed dentition?	Dental inclusion in mixed dentition is a relatively common occurrence. The prevalence of dental inclusion varies greatly depending on the population studied and the criteria used to define inclusion. Studies have shown a prevalence range of 0.3% to 10.3% of all permanent teeth in mixed dentition undergoing active eruption. The most commonly impacted teeth are the maxillary canines, followed by the mandibular premolars and molars. It’s important for parents and caregivers to schedule regular dental check-ups for children in mixed dentition to ensure proper dental development and identify any potential issues.	5.30	2.60	0.002	<0.001
Dental Inclusion	11. How is the diagnosis for a maxillary included canine made?	The diagnosis of a maxillary included canine usually involves a clinical examination and imaging techniques such as panoramic radiography and 3D cone beam computed tomography (CBCT). The clinical examination involves evaluating the position and eruption status of the canine tooth in question, as well as its relation to adjacent teeth and the overall dental arch. Imaging techniques can provide a detailed view of the tooth’s position and orientation in the jawbone and help the dental professional determine the optimal treatment plan. Based on these findings, the dentist or orthodontist can make a diagnosis and develop a treatment plan, which may involve orthodontic therapy to create space in the arch or surgical removal of the impacted canineIn some cases, the dentist or orthodontist may refer the patient to an oral surgeon who specializes in managing impacted teeth.	4.90	2.50	0.002	0.002
Dental Inclusion	12. How can maxillary canine inclusion be prevented?	Maxillary canine inclusion, which is the failure of the canine teeth to emerge into their correct position in the upper jaw, can be prevented through the following measures: Regular dental check-ups should be conducted: Regular dental visits can help identify the signs of tooth eruption problems early on and reduce the risk of complications. Early orthodontic treatment: When a child’s teeth are developing, an orthodontist can identify any potential problems that may affect tooth eruption and bite alignment. Early orthodontic treatment can help guide the teeth into their proper position, preventing unnecessary extractions in the future. Space maintenance: It is essential to maintain sufficient space for the erupting canines. Space maintainers should be used if necessary in cases where a child may lose a primary tooth prematurely. Dental X-rays: Dental X-rays can reveal impacted teeth or other problems that aren’t visible during a visual exam. They can also confirm the position of the canine teeth, allowing for appropriate action. Surgery: In some cases, extraction of primary teeth, or the exposure of the impacted canines may be necessary to aid their eruption. Good oral hygiene: Simple oral hygiene practices like brushing and flossing can help minimize the risk of infection or inflammation which may contribute to canine tooth impaction.	4.80	2.30	0.004	0.015
Open Bite	13. What are the causes of open bite in childhood?	Open bite in childhood could be due to several reasons, including: (1) Thumb Sucking or Pacifier Use: Sustained thumb sucking, pacifier use or tongue thrusting habits can lead to an open bite. (2) Tongue Thrusting: Tongue thrusting is a condition where the tongue protrudes through the front teeth during swallowing. (3) Genetics: Children may also develop an open bite if their parents have a similar dental condition. (4) Mouth Breathing: If a child has difficulty breathing through their nose and breathes through their mouth instead, it can affect the way their teeth and jaws develop, leading to an open bite. (5) Skeletal Deficiency: A child may have an open bite due to a deficient jaw, which causes the upper and lower teeth to not meet when the mouth is closed. It’s recommended to consult with an orthodontist if your child has an open bite to determine the cause and the best course of treatment.	5.30	2.60	0.008	<0.001
Open Bite	14. Which orthodontic appliances can be used for open bite in mixed dentition?	In mixed dentition, orthodontic appliances that can be used for open bite include: Active Plate: This appliance uses acrylic to pressurize or depressurize the arch and move teeth, making it suitable for treating open bites. Habit Breaking Appliance: This appliance is used in patients with poor oral habits like thumb sucking or tongue thrusting, which can cause an open bite. Expansion Appliance: This appliance can be used to widen the upper jaw if it is too narrow, which can contribute to the development or exacerbation of an open bite. Removable Functional Appliances: These appliances are used for growth modification and bite correction, which can be useful in cases of open bite. Lip Bumper: This is a type of archwire that fits into slots on the molar bands, placing pressure on the lips’ muscles and moving the molars back. It is important to consult with an orthodontist to determine the best appliance for your specific case.	4.60	2.10	0.017	0.004
Open Bite	15. What is the role of the tongue interposition in the open bite?	Tongue interposition refers to the habit of placing the tongue between the teeth during swallowing or at rest, which can contribute to the development of an open bite. An open bite is a dental condition where the front teeth do not meet when the back teeth are closed together. The tongue can act as a pressure force and push against the teeth, which can lead to the teeth shifting and creating gaps between them. Over time, this can cause the front teeth to no longer touch when biting down, resulting in an open bite. Therefore, correcting the tongue interposition habit is a crucial part of treating an open bite. This may involve working with a speech therapist or orthodontist to correct tongue posture and movements, as well as using specific appliances to help train the tongue to rest in the correct position.	4.90	2.50	0.003	0.002
Atypical swallowing	16. What are the causes of atypical swallowing in children?	There are several possible causes of atypical swallowing in children, including: Tongue thrusting: This occurs when the child pushes their tongue forward during swallowing. Oral sensory processing disorders: These can lead to difficulties with coordination and motor planning of the muscles used for swallowing. Structural abnormalities: Structural issues, such as cleft palate, can affect the mechanics of swallowing. Neurological disorders: Certain neurological conditions may cause difficulties with swallowing, such as cerebral palsy or muscular dystrophy. GERD: Gastroesophageal reflux disease (GERD) can irritate the esophagus and cause discomfort during swallowing. Airway issues: Obstructive sleep apnea or other airway issues can affect the ability to swallow properly. It is important for children with atypical swallowing to be evaluated by a healthcare professional to determine the underlying cause and to develop an appropriate treatment plan.	4.90	2.60	0.028	<0.001
Atypical swallowing	17. Is there a correlation between atypical swallowing and phonation problems?	Yes, there can be a correlation between atypical swallowing (also known as dysphagia) and phonation problems. This is because the muscles and structures involved in swallowing are also important for producing speech sounds. Dysphagia can affect the movement and coordination of these muscles, which can result in phonation problems such as hoarseness, difficulty projecting the voice, and changes in pitch or volume. If you are experiencing any difficulties with swallowing or phonation, it is important to consult a healthcare professional for evaluation and treatment	4.80	2.70	0.055	<0.001
Atypical swallowing	18. How can atypical swallowing in childhood be solved?	Atypical swallowing in childhood, also known as orofacial myofunctional disorders, can be addressed through a variety of interventions, including: Myofunctional Therapy: This is a specialized therapy to train the muscles in the face and mouth to function correctly for swallowing and speaking. Orthodontic Treatment: Orthodontic treatment can help align the teeth and jaws, making it easier to swallow and speak. Speech Therapy: Speech therapy can help children improve their speech and language skills and learn strategies to improve their swallowing. Nutrition and Feeding Support: Children with atypical swallowing may benefit from working with a nutritionist or feeding specialist who can assess their feeding skills and offer support in developing appropriate feeding strategies. Surgery: In some cases, surgery may be necessary to correct anatomical abnormalities that are contributing to the atypical swallowing pattern. It is important to work with a qualified healthcare provider to determine the best course of treatment for your child’s specific needs.	5.40	2.80	0.001	<0.001
Deep Bite	19. Is there a correlation between deep bite and temporomandibular joint disorders?	Yes, research has suggested that there may be a correlation between a deep bite and temporomandibular joint disorders (TMD). A deep bite can create an abnormal stress and load on the temporomandibular joint (TMJ), which can lead to TMD symptoms such as jaw pain, clicking or popping sounds when opening or closing the mouth, difficulty opening or closing the mouth, and headaches. However, it is important to note that not all cases of deep bite lead to TMD, and there may be other factors contributing to the development of TMD symptoms. If you are experiencing TMD symptoms, it is best to consult with a dentist or a medical professional who specializes in TMJ disorders for proper evaluation and treatment.	4.90	2.50	0.012	0.002
Deep Bite	20. What is overbite in orthodontics?	An overbite in orthodontics refers to the vertical overlap of the upper front teeth over the lower front teeth when the back teeth are in contact. A certain amount of overbite is normal, but excessive overbite can lead to problems such as tooth wear, speech difficulties, and difficulty chewing. Orthodontic treatment can be used to correct overbite, often by using braces or other appliances to reposition the teeth and jaws.	5.10	2.30	0.036	0.015
Deep Bite	21. What are the relationships between deep bite and parafunction of the lower incisors?	Deep bite and parafunction of the lower incisors may have a complex relationship. In patients with deep bite (when the upper front teeth cover too much of the lower front teeth), the lower incisors may experience increased pressure and wear due to the contact with the upper teeth. Over time, this may lead to parafunction (abnormal function) of the lower incisors, such as grinding or clenching, as the lower jaw tries to find a more comfortable and stable position. On the other hand, parafunction of the lower incisors may contribute to the development or worsening of deep bite by putting excessive force on the lower front teeth, causing them to become more angled and create more space for the upper front teeth to overlap. It is important to address both deep bite and parafunction to ensure long-term dental health and stability.	5.10	2.50	0.017	<0.001

**Table 2:** This table shows the 7 clinical scenarios submitted to ChatGPT and the answers obtained. Then, the accuracy and completeness mean score and Shapiro–Wilk p score were calculated for each answer obtained. Clinical scenarios and answers given by ChatGPT-4.

Category	Clinical Scenario	ChatGPT-4 Answer	Accuracy Mean Score (Range 1–6)	Completeness Mean Score (Range 1–3)	Accuracy Shapiro–Wilk p Score	Completeness Shapiro–Wilk p Score
	1. CLINICAL CASE N.1. A 10-year-old patient, canine included familiarity, mixed dentition. Presence of 53 and 63 in the arch. What is the correct clinical diagnostic approach?	The correct clinical diagnostic approach in this case would be as follows: Conduct a thorough medical and dental history intake to understand the patient’s health status and dental concerns. Perform a comprehensive dental examination of the patient’s teeth, gums, jaw, and bite. Take intraoral and extraoral photographs of the patient’s teeth and face to document the existing condition. Take dental radiographs (X-rays) to evaluate the presence and location of permanent teeth, assess the extent of any dental decay or damage, and determine the position of the canines and any other impacted teeth. Based on your findings, you may refer the patient to an orthodontist for further evaluation and treatment. The orthodontist may recommend extraction of the deciduous teeth, space maintainers, or other orthodontic appliances to guide the eruption of the permanent teeth and ensure proper alignment.	4.80	2.60	0.191	<0.001
Clinical case	2. CLINICAL CASE N.2. An 8-year-old patient in mixed dentition with class III malocclusion and reported upper laterals agenesis is referred for orthodontic evaluation. What clinical and radiological aspects influence the treatment plan?	In this case, the clinical and radiological aspects that will influence the treatment plan include: Age: The patient is 8 years old which means that they are still growing and this should be taken into consideration when planning treatment. Class III malocclusion: This type of malocclusion shows a discrepancy between the upper and lower jaw, with the lower jaw positioned more forward than the upper jaw. Upper laterals agenesis: This means that the patient is missing their upper lateral incisors, which can greatly impact their smile and esthetics. Facial growth pattern: A detailed assessment of the facial growth pattern should be done to determine if the patient has a normal or abnormal growth pattern, which will influence the treatment plan. Radiographic evaluation: A panoramic radiograph is needed to assess the stage of dental development, the presence or absence of all teeth, and evaluate the position of the roots of adjacent teeth. Space analysis: Space analysis should be done to evaluate the amount of space available for the missing teeth, and whether any other teeth need to be extracted to create space. Orthodontic appliance selection: A decision must be made on what type of orthodontic appliance to use. In this case, it may be necessary to use a combination of orthodontic treatment and prosthodontic treatment to achieve optimal results. In summary, a comprehensive evaluation of the patient’s clinical and radiographic features should be done to develop an optimal treatment plan that will address not only the orthodontic but also the prosthetic aspects of the treatment.	5.00	2.50	0.010	0.002
Clinical case	3. CLINICAL CASE N.3. A 10-year-old female patient with Class I malocclusion, swallowing disorders and ankyloglossia: what is the correct diagnostic and therapeutic procedure?	The correct diagnostic procedure for a 10-year-old female patient with Class I malocclusion, swallowing disorders, and ankyloglossia would involve a thorough evaluation by an orthodontist and/or an oral surgeon. This would typically include a clinical assessment of the patient’s dental and facial features, as well as a detailed examination of the tongue and other oral structures. The orthodontist and/or oral surgeon may also request imaging studies, such as X-rays or CT scans, to better evaluate the extent of the malocclusion and other underlying conditions. Therapeutic procedures for this patient may involve a combination of orthodontic treatment to address the malocclusion, speech therapy to improve swallowing and speech, and surgical intervention to correct the ankyloglossia if warranted. Surgical intervention may involve a frenectomy, which is a simple surgical procedure to remove the frenum (the tissue connecting the tongue to the floor of the mouth) and release the tongue. This can be done using a scalpel, scissors or laser surgery technique. Speech therapy can help identify and correct swallowing and speech disorders through exercises that improve the coordination of oral muscles while eating or speaking. Orthodontic treatment may range from simple appliances like plates, functional appliances to correct the Class I malocclusion. It is important to work with a team of specialists to ensure that the patient receives comprehensive and effective care for these conditions.	5.20	2.70	<0.001	<0.001
Clinical case	4. CLINICAL CASE N.4. 7 year old male patient with increased overjet and proclined incisors. What is the clinical approach to resolve the malocclusion?	The clinical approach for resolving an increased overjet and proclined incisors malocclusion in a 7-year-old male patient may include: Observing the patient’s growth and development—In some cases, the malocclusion may self-correct as the patient’s jaw develops. Interceptive orthodontic treatment—The patient may benefit from early orthodontic intervention to guide the growth of the jaw and adjust the position of the teeth. Habit correction—If the malocclusion is caused by thumb sucking, tongue thrusting, or other habits, working on correcting those habits may help resolve the issue. Extraction of primary teeth—In some cases, the premature loss of primary teeth can lead to an increased overjet. Extracting the primary teeth may create space for the permanent teeth to come in properly. Braces or clear aligners—Once the patient has all of their permanent teeth, traditional braces or clear aligners may be used to correct the position of the teeth. It is important to consult with an orthodontist to determine the best course of action for each individual patient.	4.50	2.30	0.052	0.015
	5. CLINICAL CASE N.5. Uncooperative patient: 7 years old child, multiple caries, malocclusion Class II 1st division. Which is the best treatment plan?	The best treatment plan for an uncooperative 7-year-old child with multiple caries and malocclusion Class II 1st division may vary based on the severity and extent of their condition. However, generally, the following treatment plan may be considered: Behavior management: Prior to starting any dental treatment, the child’s behavior should be managed effectively. Behavior management techniques can include positive reinforcement, modeling, tell-show-do, and humor. Oral hygiene instruction: Education about proper oral hygiene practices should be provided to the child and their caregiver. This can include brushing techniques, flossing, and the use of fluoride. Caries management: Depending on the severity and extent of the caries, the child may require fillings, and/or extractions. In more severe cases, pulp therapy or crowns may be needed. Orthodontic treatment: To correct the malocclusion Class II 1st division, the child may require orthodontic treatment. This can include braces, headgear, or functional appliances, depending on the severity of the malocclusion. Preventive therapy: Following treatment, the child should receive preventive therapy, including regular dental check-ups, fluoride treatment, and sealants. It is important to note that the overall success of the treatment plan will depend on the child’s cooperation, as well as the caregiver’s involvement in helping them to maintain good oral health habits.	5.20	2.60	0.011	<0.001
	6. CLINICAL CASE N.6. 8-year-old child with oral respiratory disease, hypertrophic adenoids, narrow palate and open bite. What is the diagnostic and therapeutic approach to follow?	The diagnostic approach for an 8-year-old child with oral respiratory disease, hypertrophic adenoids, narrow palate, and open bite would typically involve a thorough physical examination, medical history review, and diagnostic testing. The physical examination would likely include an evaluation of the child’s airway, including the size and position of the tonsils and adenoids. The dentist or pediatrician would also evaluate the child’s teeth, jaw, and palate for any signs of malocclusion or other orthodontic issues. In terms of diagnostic testing, the child may undergo a polysomnogram or sleep study to evaluate their breathing during sleep. Imaging tests such as a CT scan or MRI may also be necessary to provide a comprehensive evaluation of the child’s airway and related structures. The therapeutic approach for this child would depend on the severity of their condition and the underlying cause(s) of their symptoms. Treatment may involve a combination of medical interventions to manage the respiratory disease and orthodontic treatment to correct any dental or jaw alignment issues. Medical interventions may include medications to reduce inflammation or relieve symptoms, allergy testing and treatment, or surgery to remove enlarged adenoids or tonsils. Orthodontic treatment may include using braces or other appliances to correct the child’s malocclusion and improve jaw alignment. In some cases, an interdisciplinary approach involving specialists such as a dentist, pediatrician, and ear, nose, and throat specialist may be necessary to provide optimal care for the child’s condition.	5.30	2.50	0.008	<0.001
	7. CLINICAL CASE N.7. Dental emergency: A 9-year-old boy falls in a swimming pool and fractures his upper incisor. He also has mobility of the lower incisor group. What is the best treatment?	It is important to seek dental attention as soon as possible. Depending on the severity of the fracture and the extent of the mobility, the dentist may perform a root canal treatment or an extraction. It is important to save the natural tooth if possible. The mobility of the lower incisor group also needs to be evaluated and treated accordingly. In the meantime, the child should avoid hard foods and contact sports to prevent further damage to the teeth.	4.50	2.20	0.083	0.025

## Data Availability

The raw data supporting the conclusions of this article will be made available by the authors on request.
